# Is sex necessary for the proliferation and transmission of *Pneumocystis*?

**DOI:** 10.1371/journal.ppat.1007409

**Published:** 2018-12-06

**Authors:** Philippe M. Hauser, Melanie T. Cushion

**Affiliations:** 1 Institute of Microbiology, Lausanne University Hospital, Lausanne, Switzerland; 2 Department of Internal Medicine, University of Cincinnati College of Medicine, Cincinnati, Ohio, United States of America; 3 Veterans Administration Medical Center, University of Cincinnati College of Medicine, Cincinnati, Ohio, United States of America; McGill University, CANADA

The species within the genus *Pneumocystis* constitute a group of fungi that colonize mammalian lungs. Should the host’s immune system become weakened, this colonization can transform into a lethal pneumonia. Each *Pneumocystis* species appears to manifest a strict specificity for a single mammalian species, though there may be a few exceptions. The lack of an established long-term method of in vitro cultivation for these fungi has historically hindered their study. However, the advent of high-throughput methods allowed sequencing of the genomes of *P*. *jirovecii*, *P*. *carinii*, and *P*. *murina* that infect, respectively, humans, rats, and mice [1,2]. The analysis of these genomes and their transcriptomes revealed the absence of several metabolic pathways, demonstrating their obligate parasitism and requirement to scavenge compounds from their hosts [3–5]. Furthermore, they are biotrophic parasites because they do not kill host cells and have their sex cycle within their host [6–8]. The life cycle of these fungal pathogens remains elusive as a consequence of the absence of the inability to propagate them outside of the mammalian lung. However, comparative genomics, transcriptomal, and whole-genome analyses recently brought crucial new insights into the *Pneumocystis* life cycle. In this review, we update the life cycle in light of these new findings.

## What is the currently proposed *Pneumocystis* life cycle?

**Fig 1:**
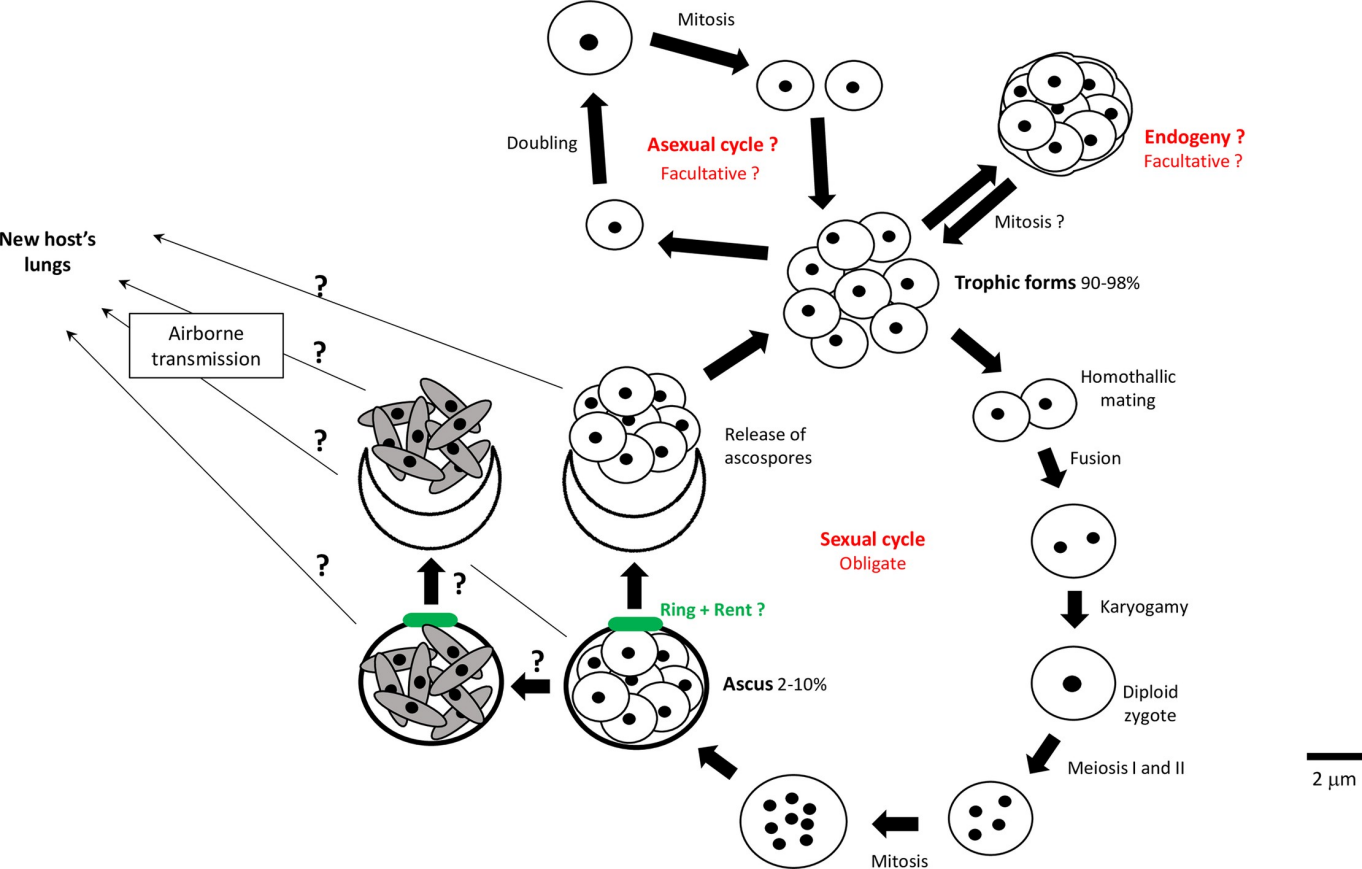
Hypothetical life cycle of *Pneumocystis*. Black dots represent nuclei. The question marks indicate events not or poorly supported by the data [9–11]. Elongated ascospores that present a condensed cytoplasm are shown as gray spindle-shaped cells.

**Fig 2:**
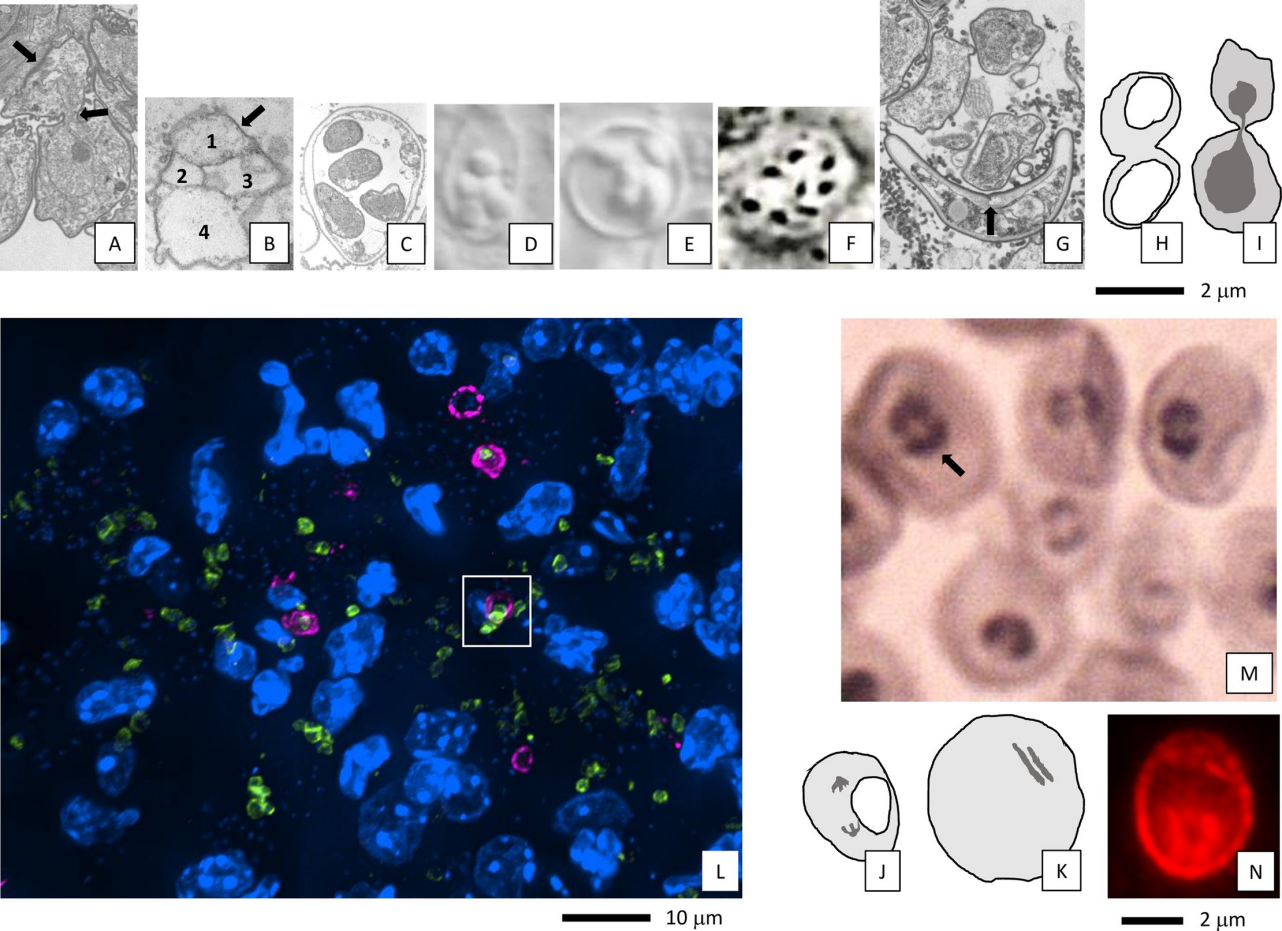
Microscopic observations of *Pneumocystis*. (A, G) Cushion’s data, electron micrsocopy (except [D] and [E] [differential interference contrast], and [F] [Diff-Quik staining, bright field]). (A) A trophic form tightly interdigitated with the host’s alveolar epithelial pneumocytes type I (top arrow). This trophic form also shows fusion with a second trophic form at the level of the cell membrane as well as possibly at the level of the nuclear membrane (middle arrow). (B) Endogeny with four daughter cells within a trophic form. The daughter cells are numbered on the image. Note the absence of cell wall (arrow). (C, D) Rounded ascopsores within an ascus. (E, F) Elongated and condensed ascopsores within an ascus. (G) Collapsed crescent ascus with rounded trophic forms associated. The latter correspond supposedly to released ascopsores. A rent in the ascus cell wall is highlighted by the arrow. (H) Dividing or mating trophic forms; the white rounded structures are supposedly a vacuole (drawn from the electron microscopic image of Fig 4 of [13]). (I) Supposedly mating trophic forms showing fusion of nuclei in dark gray suggesting karyogamy (from Fig 3 of [13]). (J) Karyokinesis within a trophic form; the spindles poles are in dark gray; the white rounded structure is supposedly a vacuole (from Fig 5 of [9]). (K) Synaptonemal complex shown in dark gray in a young ascus (from Fig 2 of [16]). (L) *P*. *murina* cell population from resected infected lungs. Green, anti-p57 surface glycoprotein specific to ascospores; magenta, anti-glucan; blue, DNA stained with DAPI. An ascus putatively releasing ascopsores is shown within the boxed area (adapted from [23]). (M) Parentheses-like structures on *P*. *jirovecii* asci. The arrow points to this structure of a single ascus. Grocott’s methenamine silver staining (Institute of Microbiology, Lausanne University Hospital). (N) Ascus stained with an antibody conjugated to Alexafluor 594 raised against 1,3-β glucan featuring a circular structure [15]. Alexafluor 594, fluorescent dye; Diff-Quik, modified Giemsa stain; p57, surface glycoprotein with a molecular weight of 57 kDa.

In the absence of direct observation of proliferating *Pneumocystis*, the proposed life cycles were largely derived from microscopic images of rodent or human infected lungs or of short-term cultures in vitro [9–11]. The commonly proposed life cycle typically includes both asexual and sexual cycles (Fig 1), and no difference among the different *Pneumocystis* species has been suggested. Two main cellular forms were identified during the infection, though many intermediate stages have been recognized: the trophic forms, which are pleomorphic in shape, devoid of a cell wall, haploid, and the most abundant of the life cycle stages (90% to 98% of the population); and the asci, which are rounded to ovate, are endowed with a thick cell wall, contain eight daughter cells, and are typically low in abundance (2% to 10%). As obligate parasites, the entire life cycle takes place within the host’s lungs. Unlike many other parasites, these fungi maintain an extracellular existence in the alveoli. Infrequent intracellular localization have been reported [9], but the significance remains difficult to assess. The trophic forms tightly interdigitate with the host’s alveolar epithelial pneumocytes type I (Fig 2A), whereas asci are primarily localized within the alveolar lumen. Trophic forms are thought to reproduce asexually by binary fission, and perhaps by “endogeny,” a reproductive process that remains poorly characterized and has been reported in only two studies [9,11]. Endogeny would generate a large trophic form containing a varying number of smaller trophic forms (Fig 2B). The trophic forms are also implicated as mating partners that enter into the sexual cycle involving meiosis followed by one mitosis that culminate in the production of asci—each containing eight daughter cells, the ascospores. The ascospores have been described as rounded (Fig 2C and 2D) or elongated in shape (Fig 2E and 2F). Most morphological studies reported collapsed and empty asci with a crescent shape with rounded ascospores that, supposedly, remained close to the asci surface after their release (Fig 2G). Release of elongated forms was purportedly observed with phase contrast microscopy [12]. Other features shown in Fig 1 are discussed in the following sections.

## Is there really an asexual cycle within the *Pneumocystis* life cycle?

The occurrence of an asexual cycle is difficult to assess because trophic cells dividing or fusing upon mating often cannot be differentiated by microscopic images (Fig 2H). Such events were reported in only two studies [9,13]. The trophic forms sometimes presented fusion at the level of the cell membrane as well as possibly at the level of the nuclear membrane (Fig 2A and 2I), suggesting karyogamy during mating rather than karyokinesis during mitosis (i.e., dividing nucleus, a hallmark of mitosis). Karyokinesis within a trophic form was reported only once [9] (Fig 2J). However, it should be noted that most ultrastructural studies published examples of a given life cycle stage, and quantitative studies have not been conducted. Based on the ratio of the number of trophic forms to that of empty asci in short-term cultures, the asexual cycle was proposed to not occur because sex was sufficient to account for the proliferation observed [14]. However, reliance upon such an imperfect culture method could have influenced the proliferative capacity of the fungi accounting for this lack of asexual replication. New data presented below warrant a reexamination of the asexual cycle, which could temporally vary in mode and frequency.

## Does the asexual cycle contribute to the proliferation of *Pneumocystis*?

Interestingly, inhibition of the sexual cycle using echinocandins that target 1,3-β glucan synthesis, and thus the production of asci cell walls, also inhibited the growth of the trophic forms, though to a lesser degree [15]. One could posit that the asexual cycle is linked to the sexual cycle through regulatory factors or that trophic forms harbor small amounts of 1,3-β glucan that render them sensitive to echinocandins. Whatever the reason, trophic forms seem to have remained in a latent state during the echinocandin treatment because withdrawal of the drug led to repopulation of asci within a week. Therefore, trophic forms may have subtle roles within the life cycle such as ensuring survival of the fungus rather than proliferation.

## Is there really a sexual cycle within the *Pneumocystis* life cycle?

The observation of synaptonemal complexes involved in the alignment of homologous chromosomes during meiosis within young asci first suggested that a sexual cycle occurs [16] (Fig 2K). This was further supported by the identification of the receptor to the minus sexual pheromone [17] and of the meiotic control pathway [18]. Recently, comparative genomics identified three *Pneumocystis* genes involved in the cellular differentiation necessary for mating and entering into the sexual cycle [19]. They constitute a single mating locus controlling both mating types—plus and minus—suggesting that the mode of reproduction of *Pneumocystis* species is homothallism, i.e., self-fertilization from a single cell without the need of a compatible partner. The frequent concomitant expression of these three genes during human infections suggested that sexuality is obligatory to complete the life cycle [20]. Transcription of the genes involved in mating and meiosis during infection has been evidenced also in rat and mice [2]. Obligate production of asci would be consistent with their presence in the vast majority of, if not all, infections as well as with their essentiality for airborne transmission of the fungus [15,21]. Obligate sexuality is also compatible with the reported activation of sex-related genes upon treatment with echinocandins in RNA-sequence analyses, as overall growth was impeded [22]. When used prophylactically, the echinocandins could prevent the development of infection and subsequent pneumonia, further supporting the requirement for sexual replication, which is inhibited by these drugs [15]. The obligate nature of *Pneumocystis* sexuality may require a secure and quick commitment of the trophic forms to sex after initiation of the infection or after release from asci. The environmental stimuli for sexual reproduction may differ from nutrient deprivation as in most fungi. Sex might even be constitutively induced in trophic forms.

## Is sex necessary for the proliferation of *Pneumocystis*?

Targeted staining of a glycoprotein specific to the surface of *P*. *murina* ascospores recently provided direct evidence that asci do release these forms during infection and that the latter are then widely present within the population (Fig 2L, green label) [23]. Thus, dehiscence, i.e., the release of the ascospores, does occur frequently within the lungs where the asci have been produced, demonstrating that sex contributes significantly to the proliferation of *Pneumocystis* (Fig 1).

## Which cycle is necessary for the transmission of *Pneumocystis*?

Inhibition of the sexual cycle using echinocandins in rodent models resulted in the lack of transmission of the infection [15], whereas in a separate study only purified asci could transmit the disease [21]. These observations strongly suggested that sex is necessary for transmission. The few published electron micrographs indicated that the ascospores are released through a simple rent in the ascus cell wall that did not show any special differentiation (Fig 2G, arrow). *Pneumocystis* asci are unitunicate, i.e., have a single layer of wall, and present a localized thickening of the wall forming an incomplete ring known as the parentheses-like structure (Fig 2M). The latter corresponds perhaps to the circular structure featured by an ascus stained with a fluorescent antibody to 1,3-β glucan (Fig 2N). The parentheses-like structure may match the ascomycetous-thickened ring in the center of which a rent is formed upon contact with humidity, allowing dehiscence [24, http://www.hiddenforest.co.nz/fungi/class/ascomycotina.htm]. This ring may function as a pressure valve that expands and lets the ascospores shoot out. This rapid deployment could explain the paucity of images or observations regarding this process. However, fungi display a large variety of dehiscence mechanisms, so that one cannot rule out that a specific opening lid—an operculum—is in fact present. Whatever the mechanism, dehiscence occurs within the lungs and contributes to proliferation, as Fig 2L strongly suggests (see above). This does not exclude that a proportion of the asci can leave the lungs through the airborne route in order to dehisce in the lungs of a new host. The asci wall is rich in β-glucans and mannans, and melanin is present in both asci and ascospores, suggesting that these factors could provide protection from environmental insults, such as UV and desiccation [25]. Numerous studies reported asci containing elongated ascospores that presented a condensed cytoplasm (Fig 2F). Such condensation also may be associated with resistance to physical insult. It is currently unclear whether elongated ascospores are present in all asci at the conclusion of the maturation or only in a proportion of the asci through a specific maturation intended for aerial transportation (Fig 1). Transmission by asci rather than ascospores would provide advantages such as genetic diversity that might be of importance for initiation of infection, as well as a quick initiation of infection owing to the eight daughter forms.

## How do you explain infections with a vast majority of trophic forms?

A few such infections were reported in athymic rats and humans [26]—rats reconstituting immunity after pneumonia [27] and human cases after breakthrough of antifolate prophylaxis [28]. These trophic replete infections were suggested to result from proliferation due to asexual multiplication because release from asci could not account for such high numbers of trophic forms. These observations suggest that the asexual cycle might be increased or activated under certain circumstances. Other circumstances in which this may happen could be during early infection due to nutrient abundance or colonization rather than active infection because of effective host immunity.

## Conclusion

The answer to the question raised in the title of the present review is most probably yes: the data gathered so far strongly suggest that sex is necessary for both proliferation and transmission of *Pneumocystis*. The asexual cycle by binary fission, and/or possibly by endogeny, might be facultative, i.e., predominate under certain conditions or during certain phases of the infection. The trophic cells might also be capable of latency crucial for the survival of the fungus. However, these possibilities cannot be presently proved or disproved in a definitive way. Important questions about the *Pneumocystis* life cycle remain: does reproduction by endogeny occur and contribute to proliferation? How do echinocandins inhibit the asexual cycle? Are the mating trophic forms of the minus type, plus type, or both? Are there different fates for asci containing elongated or rounded ascospores? What are the transmission propagules: the rounded ascospores, the elongated ascospores, the asci containing the rounded ascospores, the asci containing the elongated ascospores, or all four?
